# Utilizing Virtual Exchange to Sustain Global Health Partnerships in Medical Education

**DOI:** 10.5334/aogh.3179

**Published:** 2021-03-08

**Authors:** Ayesha Mirza, Liu Gang, Thomas Chiu

**Affiliations:** 1 Department of Pediatrics, University of Florida, College of Medicine, Jacksonville, Florida; 2 National Medical Center, Beijing Children’s Hospital, Capital Medical University, Beijing, China

## Abstract

Integrating global health (GH) training in medical education has become prevalent in the United States over the last two decades. Many medical school graduates participate in some type of international learning experience during their undergraduate/graduate training, with plans to make this a part of their life-long learning experiences. Recognizing this trend, many pediatric national organizations, such as the American Academy of Pediatrics, the Association of Pediatric Program Directors, and the American Board of Pediatrics, have developed initiatives integrating GH education into existing curricula. We report our experience with using virtual learning on a cloud-based platform to remain connected with our GH training partners, and utilize this opportunity to further strengthen our existing relationships during the ongoing COVID-19 pandemic. Overall, our experience thus far shows that this is an effective way to maintain communication even when international travel is not possible. It allows for the ongoing exchange of ideas and the development of long-term sustainable relationships. There are many important lessons our trainees can learn from such partnerships.

## Background

Integrating global health (GH) training in medical education has become prevalent in the United States over the last two decades. The increase in this trend is multifactorial [1,2,3]. Major contributing factors include, but are not solely limited to: increased international travel, increased migration, and the rapid dissemination of information with digital technology and social media. The spread of Ebola virus in the United States in 2014 following the epidemic in Liberia, Africa, in addition to the ongoing coronavirus pandemic, is a stark confirmation of globalization of disease. Hence the necessity to incorporate global health education (GHE) in graduate medical education training.

Many medical school graduates participate in some type of GHE during their undergraduate/graduate training, and wish to make this a part of their life-long learning experiences. Recognizing this trend, many pediatric national organizations, such as the American Academy of Pediatrics, the Association of Pediatric Program Directors, and the American Board of Pediatrics, have developed initiatives integrating GHE into existing curricula [4,5].

Multiple post-graduate medical education training programs across the country now offer GHE, which may take the shape of international electives, dedicated global health tracks (GHT), and/or courses.

## Our Experience

Our training program is located within a stand-alone tertiary care children’s hospital affiliated with a large academic university. Our international partner institution is a large (1000 bed) dedicated tertiary care children’s hospital, also with an academic affiliation. Primary trainees within both our institutions are pediatric residents and fellows.

We established a GHT in our program in 2019. Building on our existing relationships with collaborators in institutions in South America and Asia, the GHT was a natural segue to our preexisting global health electives initially established in 2017, in collaboration with our educational partners in Bogota, Colombia, and subsequently Beijing, China.

What is somewhat unique about our program, however, is that our GH training sites are not in rural/underserved locations, as is the case with many GH experiences; rather as mentioned previously, these are large tertiary care academic centers in their respective countries. Through these elective opportunities, we already had a successful bi-directional exchange of trainees (residents and fellows) with our partners in Bogota and were set to send trainees to Beijing in the spring of 2020. However, by this time we were in the thick of the Coronavirus Disease 2019 (COVID-19) pandemic, and everything came to a grinding halt. We did not anticipate, neither were we prepared, for the effect that a natural disaster such as a global pandemic could have on these exchange programs, given that a culture of collaboration and effective communication is the cornerstone of international partnerships.

As COVID-19 continued to rage on, we struggled with how to continue fostering these relationships that had taken so much time and trust to build. In an effort to maintain communication and continue to build a collegial relationship, we brainstormed with our colleagues in Beijing and decided to experiment with using virtual learning for clinical case discussions and exchange ideas on a monthly basis.

We agreed to start these discussions at 7:30 a.m. Eastern Time (7:30 p.m. in Beijing), a time that was reasonable for both parties involved. The case discussions were conducted via Zoom and were in English. Links were sent out to all participants a few weeks before the actual case discussion. The meeting started with a PowerPoint presentation of the case to be discussed followed by a Q&A session. The cases included management of opportunistic infections in immunocompromised children, methicillin resistant staphylococcal infections, and management of tuberculosis. We concluded with some final discussion points summarizing the case and lessons learnt. Given that we were in the midst of a pandemic, we had some informal discussions about the treatment of COVID-19 cases; however, because these were being managed in separate hospitals designated specifically for this disease entity in our partner institution city, we have thus far not had any formal case discussion on this topic.

To date we have had four educational exchange meetings via Zoom and have three others planned through the end of the year. The number of participants at each meeting has grown as more people have become aware of this exchange, going from a total of five participants at the very first meeting to 25 by the fourth meeting. The participants have included residents as well as senior and junior faculty at both institutions.

The feedback we received from both sides thus far has been extremely positive. Feedback was obtained through both informal discussion as well as a brief survey using mostly yes/no responses, whereby respondents assessed the usefulness of the discussions, format utilized, challenges encountered, and future directions (attachment). Initial challenges we faced were the different international time zones, some difficulty with connectivity over the platform, and hesitancy in communication due to language barriers. In order to mitigate the language barrier to some extent, after the first two sessions, PowerPoint slides were shared at least a week before the actual meeting to allow for case review and give more time to assimilate the information.

By far all participants felt that these virtual discussions, particularly at a time when no travel or face-to-face meetings were possible, have been very helpful. Most felt that the use of cloud-based technology as a platform for these presentations also worked well. A small minority did experience some technical difficulties with logging in or audio, but these were quickly resolved. Participants also expressed the desire to have small didactic sessions interspersed with clinical case discussions. Several have suggested topics for future review and collaboration, including comparison of different disease management strategies based on local resources in both countries (China/U.S.). While language was considered somewhat of a barrier for some, others felt that this also gave them an opportunity to improve their English language skills.

Participants on our side expressed enthusiasm and support for ongoing discussions and the incorporation of short didactic learning topics in addition to clinical cases.

Below was the group comment from our educational partners in Beijing:

We have gained a lot from the online international case exchange, and the current frequency is appropriate. PPT (PowerPoint) before discussion is very helpful, and we have learned the way of case report during the process. Difficulties include language problems and lack of professional experience.

Advice: 1. Case discussion is effective in terms of form. It is very helpful to have case summary and PPT before online discussion. 2. Written summary of key issues discussed.

## Future Directions

It is evident from our experience thus far that collaboration of this nature has many benefits. It allows us the opportunity to exchange ideas and better understand challenges that may be faced in different healthcare environments, while at the same time learning how things may be done differently based on available resources. This type of experiential learning is crucial for our trainees as they learn to work across international boundaries and begin to understand the broader meaning of GH. It also serves to emphasize the importance of understanding/accepting why things may be done differently based on local context, that ‘our’ way isn’t necessarily always the right way. In a strange and unexpected twist of fate, COVID-19 also seems to have leveled the playing field in terms of equity where we (in the U.S.) now find ourselves in the unenviable position of being at the epicenter of the pandemic. We are learning not just about the intricacies involved in fostering true collaboration with colleagues in other parts of the world, but also cultural humility and respect.

At a time when educational programs may be facing budget shortfalls with declining clinical revenues, connecting virtually across the globe is also an affordable and sustainable way of fostering such programs.

There are challenges that must be acknowledged, however. Aside from issues with technology, organizing such programs, ensuring sustainability, and engaging faculty to dedicate time may seem trivial, but can present real obstacles.

We hope to continue to build on our existing program by developing ongoing case vignettes and small structured didactics, even as we hope to travel and meet with our colleagues, at a not-too-distant time in the future when it is safe to do so. Other potential ideas down the line could be to institute a morning report focused on global health, perhaps on a quarterly basis with all trainees, although logistics such as optimal timing would need to be worked out.

Cloud-based technology has shown us how to build on existing GH relationships, ensure that they are sustainable, and that they can be further strengthened by maintaining communication even in challenging times such as these.

## Additional file

The additional file for this article can be found as follows:

10.5334/aogh.3179.s1 Survey Tool. Feedback from participants regarding virtual exchange.

## Abbreviations

GH: Global Health

GHE: Global Health Education

GHT: Global Health Track

COVID-19: Coronavirus Disease 2019
