# Supplementary material for: Utilizing Virtual Exchange to Sustain Global Health Partnerships in Medical Education
Source: Ann Glob Health. 2021 Mar 8;87(1):24. doi: 10.5334/aogh.3179 (PMC7954178; doi:10.5334/aogh.3179)
Supplement: Survey Tool. — Feedback from participants regarding virtual exchange. [file agh-87-1-3179-s1.pdf]

The purpose of this questionnaire is to assess the effectiveness of the educational didactic sessions between the University of Florida/Wolfson Children's Hospital and Beijing Children's Hospital infectious disease divisions. Please respond to the questions and provide as much detail as possible. This will help us in insuring that this exchange continues to be a useful learning tool for our participants.

1. How did you learn about this clinical case based infectious diseases conference between Wolfson Children's Hospital (Jacksonville, Florida) and Beijing Children's Hospital (Beijing, China)?
   a. Department email
   b. Colleagues
   c. Other-------------------------------------

2. How many times have you participated in this case discussion over the past 3 months?
   a. Once
   b. Twice
   c. Greater than 2 times

3. How useful did you find the discussion and exchange of ideas?
   a. Very
   b. Somewhat
   c. Not useful

4. Did you experience any technical difficulty using the cloud based platform?
   a. Yes
   b. No

5. If yes to Q 4 was the problem easy to resolve?
   a. Yes
   b. No
   c. Other----------------------------

6. Do you think this kind of international collaboration is a good idea?
   a. Yes
   b. No

7. Do you feel that this exchange contributed to your individual education?
   a. Yes
   b. No

8. Do you think this type of exchange was helpful in your ability to take care of your patients?
   a. Yes
   b. No
   c. Somewhat

9. Did you find that language was a barrier to communication?

a. Yes
b. No
c. If yes how was this resolved--

10. Is there anything you would like to see done differently in future discussions?
    a. Yes
    b. No
    c. If yes what suggestions for improvement do you have -------------------------------

    ----

11. Any future topics you would like to see discussed?

____

12. Would you like any didactic talks to be part of this education exchange?

    a. Yes
    b. No
    c. If yes, are there any particular topics you would like discussed:
